# Supplementary material for: Early detection of occupational stress: Enhancing workplace safety with machine learning and large language models
Source: PLoS One. 2025 Jun 2;20(6):e0323265. doi: 10.1371/journal.pone.0323265 (PMC12129211; doi:10.1371/journal.pone.0323265)
Supplement: S5 Text — (PDF) [file pone.0323265.s005.pdf]

DEMOGRAPHIC CODE

EDUCATION LEVEL
1 = No schooling
2 = Did not complete primary school
3 = Completed standard 6
4 = Completed form 3
5 = Completed form 5
6 = Completed form 6/ certificate/ diploma
7 = Completed a bachelors degree
8 = Completed a masters degree
9 = Completed a doctoral qualification

HOUSEHOLD INCOME
1 = Less than RM2,500
2 = RM2,500-RM3,169
3 = RM3,170-RM3,969
4 = RM3,970-RM4,849
5 = RM4,850-RM5,879
6 = RM5,880-RM7,099
7 = RM7,110-RM8,699
8 = RM8,700-RM10,959
9 = RM10,960-RM15,039
10 = RM15,040 or more

SPERM QUALITY
1 = Normal
2 = Oligozoospermia
3 = Asthenozoospermia
4 = Teratozoospermia
5 = Oligoasthenozoospermia
6 = Asthenoteratozoospermia
7 = Oligoasthenoteratozoospermia
8 = Azoospermia

JOB SATISFACTION

JS1 = I feel I am being paid a fair amount for the work I do.
JS2 = Raises are too few and far between.
JS3 = I feel unappreciated by the organisation when I think about what they pay me.
JS4 = I feel satisfied with my chances for salary increases.
JS5 = There is really too little chance for promotion on my job.
JS6 = Those who do well on the job stand a fair chance of being promoted.
JS7 = People get ahead as fast as they do in other places.
JS8 = I am satisfied with my chances for promotion.
JS10 = My supervisor is unfair to me.
JS11 = My supervisor shows too little interest in the feelings of subordinates.
JS12 = I like my supervisor.
JS13 = I am not satisfied with the benefits I receive.
JS14 = The benefits we receive are as good as most other organisations offer.
JS15 = The benefit package we have is equitable.
JS16 = There are benefits we do not have which we should have.
JS17 = When I do a good job, I receive the recognition for it that I should receive.
JS18 = I do not feel that the work I do is appreciated.
JS19 = There are few rewards for those who work here.
JS20 = I don't feel my efforts are rewarded the way they should be.
JS21 = Many of our rules and procedures make doing a good job difficult.
JS22 = My efforts to do a good job are seldom blocked by red tape.
JS23 = I have too much to do at work.
JS24 = I have too much paperwork.
JS25 = I like the people I work with.
JS26 = I find I have to work harder at my job because of the incompetence of people I work with.
JS28 = There is too much bickering and fighting at work.
JS29 = I sometimes feel my job is meaningless.
JS30 = I like doing the things I do at work.
JS31 = I feel a sense of pride in doing my job.
JS32 = My job is enjoyable.
JS33 = Communications seem good within this organisation.
JS35 = I often feel that I do not know what is going on with the organisation.
JS36 = Work assignments are not fully explained.

JOB PERFORMANCE ITEMS CODE

JP4 = I find it difficult to manage work-related stress because of my fertility problem.
JP5 = My fertility problem prevent me from enjoying my work.
JP6 = I give up trying to complete specific tasks because of my fertility problem.
