# Supplementary material for: Early detection of occupational stress: Enhancing workplace safety with machine learning and large language models
Source: PLoS One. 2025 Jun 2;20(6):e0323265. doi: 10.1371/journal.pone.0323265 (PMC12129211; doi:10.1371/journal.pone.0323265)
Supplement: Attachment — Submitted filename: Response to Reviewers.pdf [file pone.0323265.s006.pdf]

March 25, 2025

Editor-in-Chief
PLOS ONE

Dear Editor-In-Chief,

We want to express our sincere gratitude for granting us the opportunity to submit the revised manuscript (Ref. No.: PONE-D-24-54716) titled "Early detection of occupational stress: Enhancing workplace safety with machine learning and large language models" to PLOS ONE. We greatly value the time and effort you and the reviewers invested in our manuscript, which has significantly improved it.

Your feedback and the reviewers' suggestions were truly invaluable. We are pleased to inform you that we have carefully addressed all the reviewers' questions and comments, making the necessary revisions to the manuscript.

Our responses to the reviewers' comments are included in the following pages, with page numbers referring to the revised manuscript that incorporates tracked changes. As the corresponding author, I confirm that all authors have reviewed the manuscript, agreed to the changes, and consented to the submission of this revised version.

We genuinely appreciate your consideration of our manuscript.
Best Regards,

Dr. Sifat Momen (Corresponding Author)
Associate Professor
Department of Electrical and Computer Engineering
North South University, Dhaka, Bangladesh
E-mail: sifat.momen@northsouth.edu

Response to The Editorial Board

Academic Editor's Comments

1. Please ensure that your manuscript meets PLOS ONE's style requirements, including those for file naming. The PLOS ONE style templates can be found at https://journals.plos.org/plosone/s/file?id=wjVg/PLOSOne_formatting_sample_main_body.pdf and https://journals.plos.org/plosone/s/file?id=ba62/PLOSOne_formatting_sample_title_authors_affiliations.pdf

   Response:

   Thank you for your guidance. We have carefully reviewed the PLOS ONE style requirements and ensured that our manuscript now adheres to all formatting and file-naming guidelines. The necessary revisions have been made in accordance with the provided templates.

2. Please note that PLOS ONE has specific guidelines on code sharing for submissions in which author-generated code underpins the findings in the manuscript. In these cases, we expect all author-generated code to be made available without restrictions upon publication of the work. Please review our guidelines at:
   https://journals.plos.org/plosone/s/materials-and-software-sharing#loc-sharing-code and ensure that your code is shared in a way that follows best practice and facilitates reproducibility and reuse.

   Response:

   Thank you for your comment. We fully support the principles of open science and reproducibility. In accordance with PLOS ONE's code-sharing guidelines, we have made all author-generated code publicly available. The complete source code underlying our findings can now be accessed via https://github.com/junayed-hasan/occupational-stress-ml/. This ensures transparency, facilitates reproducibility, and allows other researchers to build upon our work.

Note to The Reviewers

1. All modified portions in the revised manuscript are described in the blue-colored text.

2. In answering the comments, we have used the following format throughout this response letter. We first state the comments made by the respective reviewer, followed by our response.

[**Tribute**] We would like to express our deepest appreciation to the anonymous reviewers for their extremely insightful comments that greatly enhanced the quality of the paper. We have made every effort to implement the suggestions, demonstrating the effectiveness of our work.

Response to The Reviewers

Responses to the review comments are illustrated below:

Reviewer 1

Comment 1.1 – The professional English editing is recommended. The authors should get editing help from someone with full professional proficiency in English.

Response:

Thank you for your recommendation. Following your suggestion, we have thoroughly revised the manuscript, improving the language and clarity to enhance its overall readability and professionalism.

Comment 1.2 – The abstract contains a large amount of information. It is recommended to simplify the language and highlight the innovative points and main results of the study. Some important methods suggested references is higher quality or a new literature, such as CNN "DOI: 10.1109 / TFUZZ. 2024.3369944", "DOI10.3389 / fpubh. 2022.981019"; Machine learning methods can refer to "DOI10.2174/1574893614666190416152025".

Response:

Thank you for pointing this out. We have re-written the entire abstract following this suggestion. We have simplified the language, and highlighted the innovative points and main results of the study. Moreover, we have added all the three references, and some more high quality recent literature in Page 3 (Lines 64-65), and in Page 9 (Lines 266-269).

Comment 1.3 – Although SHAP and LIME are used for model interpretation, the results primarily focus on feature importance, lacking in-depth analysis of the model's decision-making process.

Response:

Thank you for the comment. Following the pointer, we have provided an in-depth analysis of the model's decision-making process for the both the "Stressed" and "Not stressed" classes in Pages 33-35 (Lines 836-863). We have provided more detailed and fine-grained analyses of the decision-making process for each class, and explained how and why the model predicts a specific class given an input.

Comment 1.4 – It is recommended to include an analysis of the differences between synthetic and real data, discuss the limitations of synthetic data generation methods, and propose improvements.

Response:

Thank you for your valuable suggestion. In response, we have significantly updated the 'External validation with synthetic data generation' section (Pages 24–28). Specifically, we have added a new subsection, 'Quality assessment of the generated data' (Pages 24–27, Lines 616–657), where we compare real and synthetic data using Principal Component Analysis (PCA) plots, Pearson's correlation heatmaps, and box plots. Additionally, we have introduced another subsection, 'Limitations and proposed improvements for synthetic data generation' (Page 28, Lines 684–702), where we discuss the limitations of the applied methods and suggest potential improvements for future research.

Comment 1.5 – High-resolution images should be provided to ensure that all details are visible and can be thoroughly examined by readers.

Response:

Thank you for your valuable comment. In response, we have significantly enhanced the quality and resolution of all figures in the manuscript. Specifically, we have improved the resolution of Figures 1–4 and added new high-quality figures and plots (Figures 10, 11, 12, 13, and 16). Additionally, we have adjusted the presentation of all figures to span the full page width, ensuring better readability and visualization.

Comment 1.6 – It is recommended to expand the literature review section, particularly with a deeper discussion of recent advancements in AI technologies for occupational stress detection.

Response:

Thank you for the recommendation. We have expanded the literature review section by adding more in-depth discussion of recent advancements in AI technologies for occupational stress in Pages 4-5 (Lines 151-178). We have also added some recent references from 2024 and 2025 to improve the section and keep the literature review up to date.

Comment 1.7 – It is recommended to add a detailed description of the synthetic data generation methods in the methodology section, including the algorithms used, parameter settings, and quality assessment of the generated data.

Response:

Thank you for your valuable suggestion. In response, we have added a new subsection, 'Synthetic data generation', under the Methods section (Pages 19–20, Lines 465–521), where we describe the four algorithms used for synthetic data generation along with their respective hyperparameter settings. Additionally, we have included a subsection, 'Quality assessment of the generated data' (Pages 24–27, Lines 616–657), comparing real and synthetic data using Principal Component Analysis (PCA) plots, Pearson's correlation heatmaps, and box plots. Lastly, we have introduced another subsection, 'Limitations and proposed improvements for synthetic data generation' (Page 28, Lines 684–702), discussing the limitations of the employed methods and potential directions for improvement.

Comment 1.8 – What is the main difference or importance of the proposed methods and the other state-of-the-arts?

Response:

Thank you for your insightful comment. In response, we have expanded the 'Comparison with existing methods' section (Page 24, Lines 604–614) to provide a more detailed analysis of how our approach differs from state-of-the-art methods. Additionally, we have introduced a new subsection, 'Methodological significance', under the Discussion section (Pages 35–37, Lines 882–904), where we elaborate on the key distinctions and advantages of our proposed methods compared to existing approaches.

Comment 1.9 – The conclusion should be concise and powerful, summarizing the main findings and contributions of the research.

Response:

Thank you for the comment. Following this, we have completely re-written the conclusions section in Pages 38-39 (Lines 958-983), making it concise, effective, and summarizing the main findings, contributions and results of the paper.

Comment 1.10 – The Conclusion section should point out the potential disadvantages and possible future research directions of the manuscript. How this work can be extended in future?

Response:

Thank you for the comment. We have discussed these in the 'Limitations and future work' subsection in Page 38, and summarized the points again in the last paragraph of the Conclusions in Page 983 (Lines 976-983).

Reviewer 2

Comment 2.1 – How does the proposed AI-based framework combine machine learning, deep learning, and large language models to create a unified approach for occupational stress detection?

Response:

Thank you for the query. The combination of machine learning, deep learning, and large language models in our AI-based framework is evident from the framework diagram in Page 12 (Figure 4), and from the computational algorithm presented in Page 14 (Algorithm 1). The machine learning models are applied on the structured tabular data with extensive data preprocessing and hyperparameter tuning to achieve the maximum performance. The 1D-CNN is applied to allow for hierarchical learning and temporal feature extraction from the data. Lastly, natural language sentences are generated from the tabular data to enable classification with domain-specific large language models, enabling domain analysis for in-depth understanding of the data domain and implications for occupational stress. In this way, the complementary benefits of each method is combined to present a unified AI-based framework for occupational stress detection with machine learning, deep learning, and large language models.

Comment 2.2 – The manuscript mentions the integration of RFECV and ANOVA for feature selection. How were these techniques combined, and what criteria were used to determine the final set of 39 critical stressors?

Response:

Thank you for this important query. We have clarified in the 'Feature selection' subsection in Page 15 (Lines 374-397 and Figure 6) that the top 28 features were selected from both RFECV and ANOVA rankings, and combined to get a total of 39 features (11 non-overlapping features). We have also explained the rationale behind selecting 28 features from each method. This process is further illustrated in the framework diagram (Page 12, Figure 4) and detailed in the computational algorithm (Page 14, Algorithm 1).

Comment 2.3 – Could you elaborate on the advanced preprocessing techniques used, particularly in managing imbalanced data or missing values?

Response:

Thank you for your query. We have clarified in the 'Dataset' section (Page 7, Line 205) that the dataset does not contain any missing values. Additionally, the data is not highly

imbalanced, as illustrated by the bar plot in Page 13 (Figure 5(b)). Therefore, no specific techniques for handling missing values or addressing class imbalance were required in this study.

Comment 2.4 – The manuscript reports a 90.32% accuracy for the ensemble model and 89.00% on synthetic data. Were other performance metrics, such as precision, recall, F1-score, and AUC-ROC, evaluated to assess the model's reliability in stress detection?

Response:

Yes, we have documented all essential performance metrics, including accuracy, precision, recall, F1-score, and AUC-ROC, across five random seeds, presenting both mean and standard deviations to ensure a thorough assessment of our models, as illustrated in Tables 3, 4, 5, 6, and 7. We have similarly documented the additional cross-validation results, as illustrated on Page 23 (Figure 10).

Comment 2.5 – It is always recommended to apply 10-fold CV for unbiased and reliable prediction results, which is not possible due to the random split of data during training and testing. It is kindly requested to consider these recommendations and add results OR discussion in the revised version with reference to the suggested references provided.

Response:

Thank you for the recommendation. Based on this, we have added the cross validation results in the 'Results' section of the paper in Page 21 (Lines 532-536) and Pages 22-23 (Lines 565-573 and Figure 10). We have analyzed the results in the 'Analysis of results' portion, comparing it with holdout validation results.

Comment 2.6 – The use of Explainable AI techniques is highlighted in the study. How were these techniques implemented, and what specific visualizations or interpretability tools were used to quantify the impact of workplace safety factors?

Response:

Thank you for this important query. We have substantially updated and clarified these in the 'Explainable AI for safety-critical feature analysis' portion of the paper in Pages 30-33 (Lines 754-835). We have added the implementation techniques and calculation details, along with more clarity through a new added figure (figure 16). To quantify the the impact

of the features, bar plots were used with normalized LIME weights and SHAP values. The full process is detailed in the manuscript.

Comment 2.7 – The study identifies excessive workload, unclear work assignments, and poor organizational communication as primary stressors. How were these percentages (e.g., 32%, 28%) quantified, and what statistical or machine learning methods supported these findings?

Response:

Thank you for another important query. Similar to the last response, the quantification method of the percentages and the techniques used are detailed in the 'Explainable AI for safety-critical feature analysis' portion of the paper in Pages 30-33 (Lines 754-835). The methods supporting these findings have also been discussed in details in the manuscript.

Reviewer 3

Comment 3.1 – In abstract, please clearly specify the Purpose, Contribution, and findings.

Response:

Thank you for the suggestion. We have re-written and updated the entire abstract to make the aim, contributions, and findings of the manuscript easily identifiable and apparent to the readers.

Comment 3.2 – Please write down the organization of the paper written at the end of Introduction.

Response:

Thank you for the suggestion. We have included the organization of the paper at the end of the 'Introduction' section in Page 4 (Lines 114-124).

Comment 3.3 – Please provide a mathematical model of proposed 1D CNN.

Response:

Thank you for the recommendation. Based on this, we have added the mathematical model of proposed 1D CNN in the 'AI models' portion of the 'Materials' subsection in Page 10 (Lines 272-288).

Comment 3.4 – Please analyze computational complexity in your proposed model.

Response:

Thank you for the comment. We have added the computational complexity of our proposed model in the 'AI models' portion of the 'Materials' subsection in Page 10 (Lines 288-308).

Comment 3.5 – It is recommended that professional proofreaders and native English corrections be used.

Response:

Thank you for your recommendation. In response, we have thoroughly revised the manuscript, making extensive edits to enhance the clarity, readability, and overall quality of English.

Reviewer 4

Comment 4.1 – It claimed that a framework has been developed with the ability to process diverse data types and provide explainable results, however, no such framework is presented in the manuscript.

Response:

We appreciate your feedback. The developed framework is evident from the framework diagram in Page 12 (Figure 4), and from the computational algorithm presented in Page 14 (Algorithm 1). The explainability of the developed framework is clearly specified in the 'Explainable AI for safety-critical feature analysis' subsection under the 'Results' section in Pages 30-35. The developed framework is also publicly deployed on the the HuggingFace platform, as reported in the 'Model deployment for workplace safety management' subsection in Pages 35-36 and Figure 19. We have also made the source codes and implementation details publicly available on github at https://github.com/junayed-hasan/occupational-stress-ml/.

Comment 4.2 – In Figure 1, the alphabetical numbering of the captions needs to be corrected.

Response:

Thank you for pointing this out. We have corrected the alphabetical numbering of the captions in Figure 1.

Comment 4.3 – Why in Figure 2, Box plots show the same range for the data distribution for six job performance survey questions?

Response:

Thank you for the query. We ave clarified this in the 'Exploratory data analysis' portion of the paper in Page 8 (Lines 247-251).

Comment 4.4 – One of the contributions is empirical demonstration by extracting more predictive features than individual methods, but a detailed explanation is missing.

Response:

Thank you for the comment. We have added the detailed explanation of the achieved results after the empirical demonstration in the 'Feature selection and elimination techniques' ablation study of Page 29 (Lines 720-740).

Comment 4.5 – More explanation of Figures 12 and 13 is required to be added.

Response:
Thank you for the recommendation. Following this, we have added detailed explanations of the two figures in Pages 33-35 (Lines 836-863, Figures 17 and 18).

Comment 4.6 – Figure 14 visibility should be increased.

Response:
Thank you for the suggestion. We have improved the visibility of the figure by scaling it up and spanning the entire pagewidth for better readability and understanding.

Reviewer 5

Comment 5.1 – The paper is well written and significantly addressed the research problem successfully by employing machine learning models and statistically analyzed results. All results are well tabulated with graphs. Also, the model has been generalized too.

Response:

We sincerely appreciate your positive feedback and kind words. We are glad that you found our work well-written and that our approach successfully addresses the research problem. Your acknowledgment of our efforts in employing machine learning models, statistical analysis, and result presentation is highly encouraging. Thank you for your thoughtful review and valuable time.
